# Risk factors for acute kidney injury after coronary artery bypass graft surgery: a systematic review and meta-analysis

**DOI:** 10.3389/fmed.2026.1722801

**Published:** 2026-02-24

**Authors:** Hongyan Chu, Shuling Li, Liyuan Cao, Guangzhe Xu, Lihua Yang, Chun Ma

**Affiliations:** 1 College of Traditional Chinese Medicine, Changchun University of Chinese Medicine, Changchun, Jilin, China; 2 Department of Geriatrics, Affiliated Hospital of Changchun University of Traditional Chinese Medicine, Changchun, Jilin, China; 3 College of Integrated Traditional Chinese and Western Medicine, Changchun University of Chinese Medicine, Changchun, Jilin, China

**Keywords:** acute kidney injury, coronary artery bypass graft, meta-analysis, risk factors, systematic review

## Abstract

**Background:**

Acute kidney injury (AKI) is one of the common and severe complications following coronary artery bypass graft (CABG) surgery, significantly increasing patient mortality, complication rates, and length of hospital stay. Although numerous studies have explored risk factors for postoperative AKI after CABG, results remain inconsistent. This systematic review and meta-analysis aim to synthesize existing evidence to identify the primary risk factors for AKI following CABG.

**Methods:**

Systematically searched PubMed, Embase, Web of Science, and Cochrane Library databases from their inception to 20 September 2025. Observational studies reporting risk factors for postoperative AKI following CABG were included. Two researchers independently performed literature screening, data extraction, and quality assessment. Random-effects models were used to calculate pooled odds ratios (ORs) and 95% confidence intervals (CIs). The I^2^ statistic was employed for heterogeneity analysis, and funnel plots and Egger’s test were used to assess publication bias.

**Results:**

A total of 17 research papers involving 33,809 patients were included. The results of the meta-analysis suggest that older age [OR = 1.05, 95% CI (1.03, 1.08)], prolonged cardiopulmonary bypass [OR = 1.14, 95% CI (1.06, 1.22)], diabetes [OR = 1.29, 95% CI (1.15, 1.45)], intra-aortic balloon pump [OR = 3.19, 95% CI (1.74, 5.85)], transfusion of red blood cells [OR = 1.73, 95% CI (1.25, 2.38)] may be associated with the occurrence of AKI after CABG.

**Conclusion:**

Meta-analysis results indicate that older age, prolonged cardiopulmonary bypass duration, diabetes, intra-aortic balloon pump use, and red blood cell transfusion are all significant risk factors for AKI following CABG. Among these, prolonged cardiopulmonary bypass duration and intra-aortic balloon pump use exert a particularly pronounced effect on AKI occurrence.

**Systematic review registration:**

[https://www.crd.york.ac.uk/prospero/], identifier [CRD420251144655].

## Background

Coronary atherosclerotic heart disease is one of the most prevalent cardiovascular conditions worldwide (1). Its incidence and mortality rates have risen steadily over recent decades, making it a significant public health threat to human health (2). With the acceleration of societal aging and changes in lifestyle, the burden of coronary heart disease has increased markedly globally (3, 4). According to World Health Organization (WHO) data, coronary heart disease has become the leading cause of death worldwide, claiming over 9 million lives annually (5). For patients with moderate to severe coronary artery disease, particularly those with multivessel disease or left main coronary artery disease, coronary artery bypass grafting (CABG) remains the primary and most effective revascularization strategy. It significantly improves myocardial ischemia, alleviates symptoms, and prolongs patient survival (6). However, as a major cardiac surgical procedure involving significant trauma and widespread impact on systemic organ function, postoperative complications remain common in CABG (7). Among these, acute kidney injury (AKI) stands as one of the most critical and clinically significant complications (8).

Acute kidney injuryis a syndrome characterized by a rapid decline in renal function within a short timeframe, manifested by elevated serum creatinine levels and/or reduced urine output. According to the KDIGO (kidney disease: Improving Global Outcomes) guidelines, AKI is diagnosed when serum creatinine increases by ≥0.3 mg/dL within 48 h postoperatively or rises ≥1.5 times the baseline level within 7 days, or when urine output falls below 0.5 mL/kg/h and persists for over 6 h (9). The incidence of postoperative AKI following CABG varies considerably across studies, ranging from approximately 5%–30%. Among these cases, approximately 1%–2% of patients require renal replacement therapy (RRT) (10). Once AKI occurs, it not only significantly prolongs hospital stays and increases medical costs but is also closely associated with in-hospital mortality and adverse long-term cardiac and renal outcomes (11). Studies indicate that inpatients with post-CABG AKI experience a 4- to 8-fold higher in-hospital mortality rate compared to those without AKI, with a significantly increased long-term mortality risk (12, 13). Even mild-to-moderate AKI exerts a persistent adverse impact on prognosis, elevating the risk of chronic kidney disease and end-stage renal disease (14). Therefore, identifying high-risk factors for post-CABG AKI and implementing early interventions hold significant clinical importance for improving patient outcomes.

In recent years, with improvements in perioperative cardiac surgical management and advances in minimally invasive techniques, the overall incidence of postoperative AKI following CABG has decreased (15). However, its impact on patient outcomes remains substantial. Multiple studies have attempted to establish predictive models or risk scoring systems to aid in the early identification of high-risk individuals (16). For instance, the Cleveland Clinic score and Mehta score are widely used in clinical practice. However, these scores are often based on specific populations, and some models fail to adequately account for the dynamic changes in intraoperative factors (17). Consequently, their predictive accuracy and external generalizability remain limited. Furthermore, substantial variations exist in study results across different regions and ethnic groups, with some risk factors even showing opposite directions across studies (18). This underscores the necessity for systematic reviews and meta-analyses to integrate existing evidence, thereby clarifying the true risk factors for postoperative AKI following CABG and their respective degrees of influence.

This study aims to conduct a systematic review to consolidate current research evidence on risk factors for postoperative AKI following CABG. It will perform a quantitative meta-analysis of major risk factors to determine their relative impact on AKI occurrence. The findings will assist clinicians in more accurately identifying high-risk patients during perioperative risk assessment, optimizing surgical strategies and postoperative management, ultimately reducing the incidence of postoperative AKI after CABG and improving patient outcomes.

## Methods

This systematic evaluation and meta-analysis will strictly follow the PRISMA (Preferred Reporting Items for Systematic Reviews and Meta-Analyses) guidelines (19). And it is registered in Prospero with registration number CRD420251144655.

### Literature search

Systematically searched PubMed, Embase, Web of Science, and Cochrane Library databases from their inception to 20 September 2025, the search terms are (“Coronary Artery Bypass”[Mesh] OR “coronary artery bypass graft” OR CABG OR “cardiac surgery”) AND (“Acute Kidney Injury”[Mesh] OR “acute kidney injury” OR AKI OR “renal failure” OR “kidney injury”) AND (“risk factor*” OR predictor* OR “associated factor*), connected using Boolean operators “AND” and “OR”. The specific search strategy is detailed in Supplementary Table 1.

### Inclusion and exclusion criteria

#### Inclusion criteria

(1) Study type: Observational studies (including cohort studies, case-control studies, and cross-sectional studies, although cross-sectional studies cannot establish temporal or causal relationships, they can still provide valuable information on associations between perioperative variables and AKI occurrence. Therefore, we included them to ensure a comprehensive evaluation of potential risk factors, while interpreting their findings with caution) are included, provided they explicitly report risk factors or associated factors for AKI following CABG surgery; (2) Study population: Adult patients (≥18 years) undergoing CABG, without restrictions on gender, region, or ethnicity; (3) Outcomes: Studies must explicitly report AKI diagnostic criteria (KDIGO, RIFLE, or AKIN criteria) and incidence, providing extractable effect size data [odds ratio (OR), risk ratio (RR), or their 95% confidence intervals (CI)]; (4) Language and time: Publications in English or Chinese, with no publication date restrictions; (5) If the same study population is used across multiple papers, include the one with the largest sample size or most complete data for analysis.

#### Exclusion criteria

(1) Non-original research, such as reviews, conference abstracts, case reports, animal studies, or mechanistic research.

(2) Studies involving subjects other than CABG patients (those undergoing combined valve replacement or other cardiac surgeries), or studies unable to distinguish data from isolated CABG procedures.

(3) Studies lacking explicit AKI diagnostic criteria or unable to extract relevant effect size data.

(4) Duplicate publications or studies with overlapping data; (5) Literature where the full text is not accessible, or studies where complete data could not be obtained despite attempts to contact the authors.

### Study selection

During the literature screening process, two researchers independently used EndNote 21 software to initially screen the literature obtained from the search, first through the titles and abstracts, and then to exclude literature that clearly did not meet the inclusion criteria. Subsequently, the remaining literature was reviewed by reading the full text in its entirety to further determine whether it met the inclusion and exclusion criteria. In case of disagreement between the two researchers during the screening process, it would be resolved through discussion and negotiation; if the negotiation still failed to reach a consensus, a third researcher would be invited to adjudicate to ensure the objectivity and consistency of the screening process.

### Data extractions

This study was conducted by two researchers who independently extracted relevant data from the eligible literature using an Excel sheet based on the inclusion criteria. The extraction included the basic information of the study (first author, year of publication, country and study design), the basic characteristics of the study population (sample size, number of AKI, gender, and mean age, Diagnostic criteria for AKI), regression analysis, In the process of data extraction, if two investigators disagreed on the data, it would be resolved through negotiation, and if no agreement could be reached, a third investigator would adjudicate to ensure the accuracy and consistency of data extraction. For each study, we preferentially extracted adjusted odds ratios (ORs) that controlled for potential confounding factors. If adjusted estimates were not available, unadjusted ORs were extracted and this was recorded.

### Quality evaluation

The risk of bias in the included studies will be evaluated independently by two investigators, and the results will be cross-checked. For cohort and case-control studies, the Newcastle Ottawa Scale [NOS (20)] will be used to assess quality. The NOS evaluates studies based on three dimensions: population selection, comparability, and exposure or outcome, with eight items totaling nine points. Scores range from 0 to 4 (low quality), 5 to 6 (moderate quality), and 7 to 9 (high quality). Studies scoring 0–4 will be excluded. For cross-sectional studies, this study will use the AHRQ quality (21) assessment tool to evaluate quality. This tool primarily assesses the rationality of the study design, the representativeness of the sample selection, the clarity of the definitions of exposure and outcome, the accuracy of the data collection process, the rationality of the statistical methods, and the completeness of the report.

### Statistical analysis

In this study, the OR and the corresponding 95% confidence interval (CI) of each included study were combined using Stata 15 software. First, for each study, we extracted the corresponding effect size OR and its 95% confidence interval. To combine these ORs, we pooled them using a random effects model, which can account for heterogeneity between studies, variability in effect sizes across studies. ORs and 95% CIs were calculated for each study and combined into an overall effect size. Heterogeneity of the model was assessed by the I^2^ statistic; if the I^2^ was greater than 50%, it was considered that there was a high degree of heterogeneity and that the sources of heterogeneity needed to be further explored. For high heterogeneity, we may conduct sensitivity analyses to identify potential factors that may affect the combined effect sizes. Asymmetry in the funnel plot indicates a higher likelihood of publication bias, which will be further evaluated using Egger’s test. *P*-value < 0.05 suggests the presence of publication bias, while a *P*-value > 0.05 suggests otherwise. If necessary, the trim-and-fill method will be used for further confirmation. The combined effect sizes will be reported as ORs and their 95% CIs to allow for interpretation of results and statistical inference.

## Results

### Literature retrieval results

**FIGURE 1:**
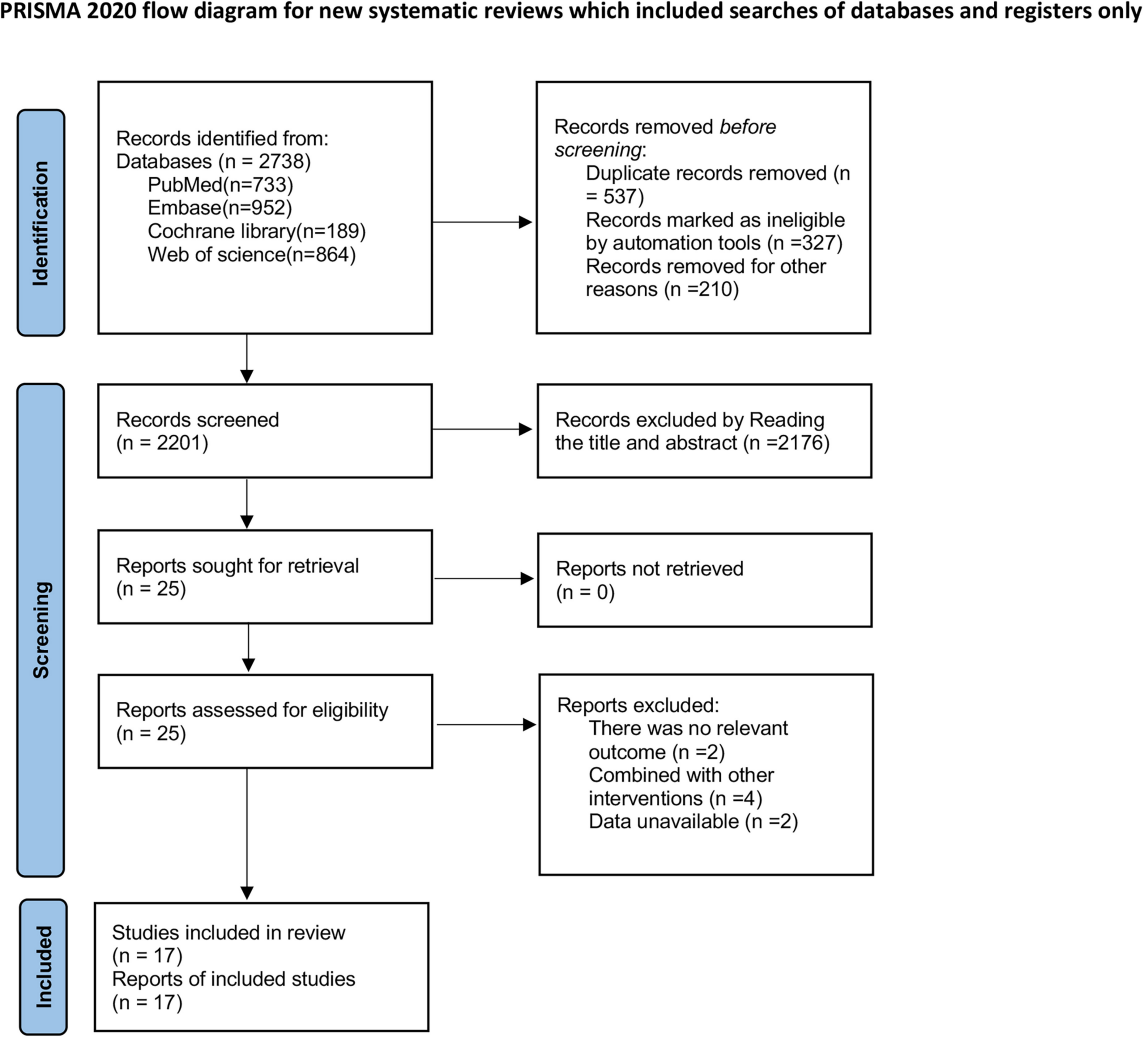
Literature search flow chart. *Consider, if feasible to do so, reporting the number of records identified from each database or register searched (rather than the total number across all databases/registers). **If automation tools were used, indicate how many records were excluded by a human and how many were excluded by automation tools Page et al. (19).

As shown in Figure 1, a total of 2,738 articles were retrieved from PubMed (*n* = 733), Embase (*n* = 952), Cochrane Library (*n* = 189), and Web of Science (*n* = 864). After removing 537 duplicate records, 2,176 articles were excluded based on title and abstract screening, and eight articles were excluded after full-text review. Ultimately, 17 studies (22–38) were included for analysis.

### Basic characteristics table of included study

This study included a total of 17 research articles (one cross-sectional study and 16 cohort studies). It involved 33,809 patients, among whom 3,283 developed AKI. The age range was 58.63–66.97 years. Detailed baseline characteristics are presented in Table 1.

**TABLE 1:** Table of basic characteristics of the included studies.

Study	Year	Study design	Country	Sample size	Gender (M/F)	Number of AKI	Mean age (years)	AKI diagnosis	Regression model
Abbasi et al. (22)		Cohort study	Pakistan	704	594/110	394	59.74	SCr increasing by 0.3 mg/dL	Logistic regression
Ahmadi et al. (23)		Cohort study	Iran	13315	90883/3347	85	58.63	Serum creatinine level 10.18 mmol/l	Logistic regression
Amini et al. (24)		Cohort study	Iran	1737	1073/664	275	60	SCr increasing by 0.3 mg/dL	Logistic regression
Aty et al. (25)		Cohort study	Australia	1914	1570/344	101	65.3	SCr increasing by 0.3 mg/dL	Logistic regression
Ay (26)		Cohort study	Turkey	79	65/14	24	61.2	Serum creatinine values above 1.5 mg/dL	Logistic regression
Barkhordari et al. (27)		Cross-sectional	Iran	3473	2374/1099	958	60.32	SCr increasing by 0.3 mg/dL	Logistic regression
Brito et al. (28)		Cohort study	Brazil	243	113/130	61	61.7	SCr increasing by 0.3 mg/dL	Logistic regression
Chen et al. (30)		Cohort study	China	2242	1327/915	219	61.5	SCr increasing by 0.3 mg/dL	Logistic regression
Chen et al. (29)		Cohort study	China	442	369/73	132	61.49	SCr increasing by 0.3 mg/dL	Logistic regression
Kim et al. (31)		Cohort study	Korea	448	330/118	34	65	SCr increasing by 0.3 mg/dL	Logistic regression
Kumad et al. (32)		Cohort study	Japan	298	200/98	47	62.9	SCr increasing by 0.3 mg/dL	Logistic regression
Kwon (33)		Cohort study	Korea	210	130/80	85	64.23	SCr increasing by 0.3 mg/dL	Logistic regression
Li (34)		Cohort study	China	907	668/2239	219	62.44	SCr increasing by 0.3 mg/dL	Logistic regression
Ng (35)		Cohort study	Singapore	1744	1500/244	391	60.89	Serum creatinine values above 1.5 mg/dL	Logistic regression
Ortega et al. (36)		Cohort study	Spain	435	244/91	54	66.8	SCr increasing by 0.3 mg/dL	Logistic regression
Yue et al. (37)		Cohort study	China	541	400/141	151	66.97	SCr increasing by 0.3 mg/dL	Logistic regression
Zhou et al. (38)		Cohort study	China	5077	4077/1000	53	63.98	SCr increasing by 0.3 mg/dL	Logistic regression

### Risk of bias results

The methodological quality of the included studies is summarized in Table 2. Among the included studies, one cross-sectional study was rated as moderate quality. For cohort studies, three studies scored seven points, six studies scored eight points, and seven studies scored nine points on the Newcastle–Ottawa Scale, indicating generally high methodological quality. In addition, the included case-control study scored nine points. Regarding specific domains, most studies demonstrated strengths in the selection domain, with clearly defined study populations and appropriate ascertainment of exposure. The outcome assessment was also generally reliable, as acute kidney injury was mostly defined using established clinical or laboratory criteria. However, the most common methodological limitation was observed in the comparability domain, where several studies did not fully adjust for important confounding variables such as baseline renal function, age, and comorbidities. This may have introduced residual confounding and should be considered when interpreting the pooled results.

**TABLE 2:** Risk bias results.

Cross-sectional											
References	Whether the source of the information is clear	Whether exposed and non-exposed groups are listed	Whether a time was given to identify patients	If not, population derived, whether the subjects were consecutive	Whether the subjective factors of the evaluator cover up other aspects of the research object	Any assessment performed to ensure quality is described	The rationale for excluding any patients from the analysis was explained	Describe measures to evaluate and/or control for confounding factors	explain how missing data were handled in the analysis	Response rates and the completeness of data collection are summarized	If there is follow-up, identify the percentage of patients with expected incomplete data or follow-up results
Barkhordari et al. (27)	Yes	Unclear	Yes	Yes	Yes	Yes	Yes	Yes	Yes	Unclear	Yes
**Cohort study**											
**References**	**Representativeness of the exposed group**	**Selection of non-exposed groups**	**Determination of exposure factors**	**Identification of outcome indicators not yet to be observed at study entry**		**Comparability of exposed and unexposed groups considered in design and statistical analysis**		**design and statistical analysis**	**Adequacy of the study’s evaluation of the outcome**	**Adequacy of follow-up in exposed and unexposed groups**	**Total scores**
Abbasi et al. (22)	*	*	*	*		**		*	*	*	9
Ahmadi et al. (23)	*	*	*	/		**		*	*	*	8
Amini et al. (24)	*	*	*	/		**		*	*	*	8
Aty et al. (25)	*	*	*	*		**		*	*	*	9
Ay (26)	*	*	*	*		**		*	*	*	9
Brito et al. (28)	*	*	*	*		**		*	*	*	9
Chen et al. (30)	*	*	*	/		**		/	*	*	7
Chen et al. (29)	*	*	*	*		**		*	*	*	9
Kim et al. (31)	*	*	*	/		**		*	*	*	8
Kumad et al. (32)	*	*	*	/		**		*	*	*	8
Kwon (33)	*	*	*	*		**		*	*	*	9
Li (34)	*	*	*	*		**		*	*	*	9
Ng (35)	*	*	*	/		**		*	*	*	8
Ortega et al. (36)	*	*	*	/		**		*	*	*	8
Yue et al. (37)	*	*	*	/		**		/	*	*	7
Zhou et al. (38)	*	*	*	/		**		/	*	*	7

*Means one score.

**Means two scores.

### Meta analysis results

#### Age

**FIGURE 2:**
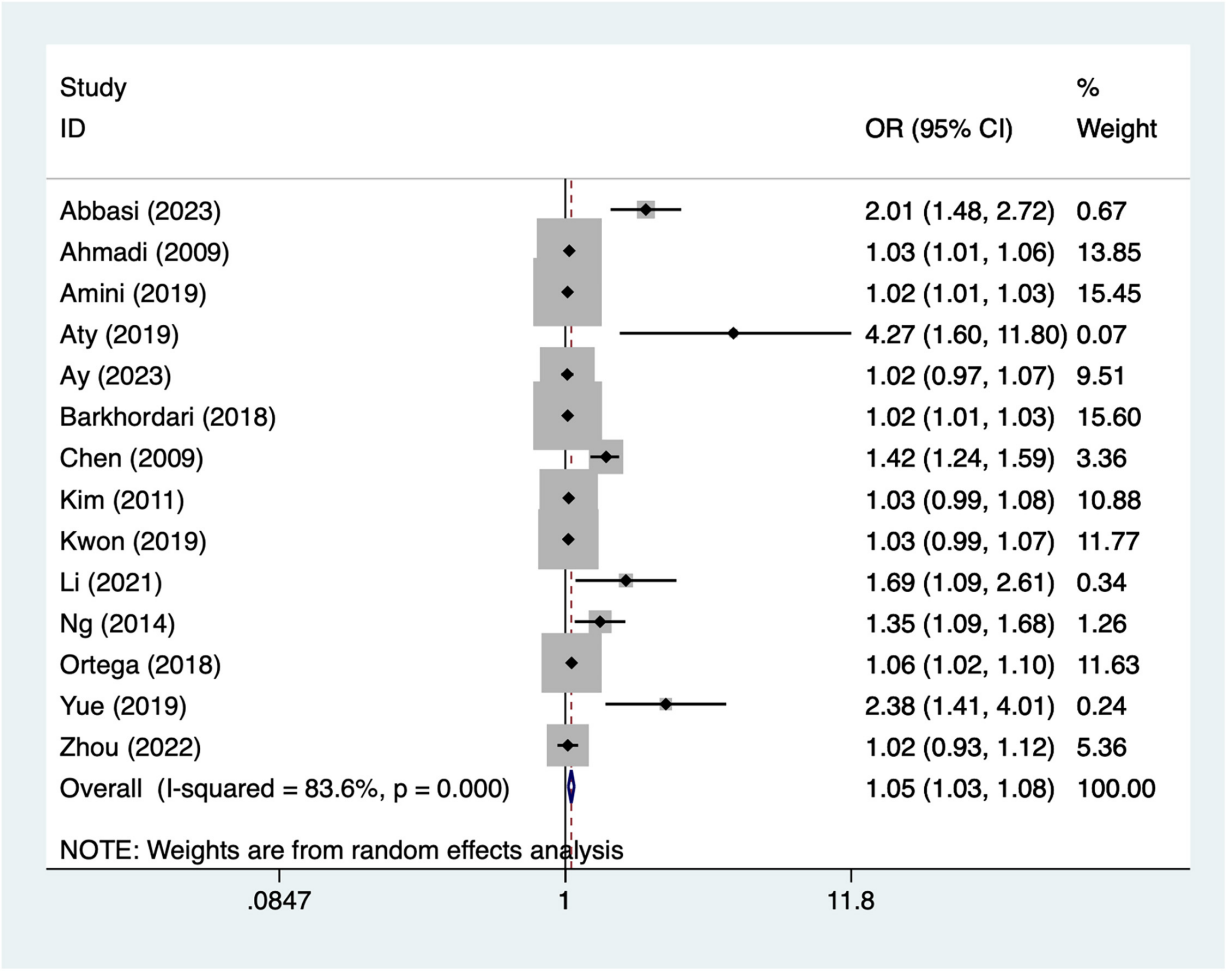
Forest plot of meta-analysis for age.

A total of 14 articles mentioned age. Heterogeneity testing (I^2^ = 83.6%, *P* = 0.001) was conducted using a random-effects model. The pooled analysis (Figure 2) indicated that older age was associated with higher odds of AKI per year increase [OR = 1.05, 95% CI (1.03, 1.08)]. Due to significant heterogeneity, sensitivity analysis was conducted by sequentially excluding individual studies. The results (Supplementary Figure 1) indicate that the indicator remains stable.

#### Prolonged cardiopulmonary bypass

**FIGURE 3:**
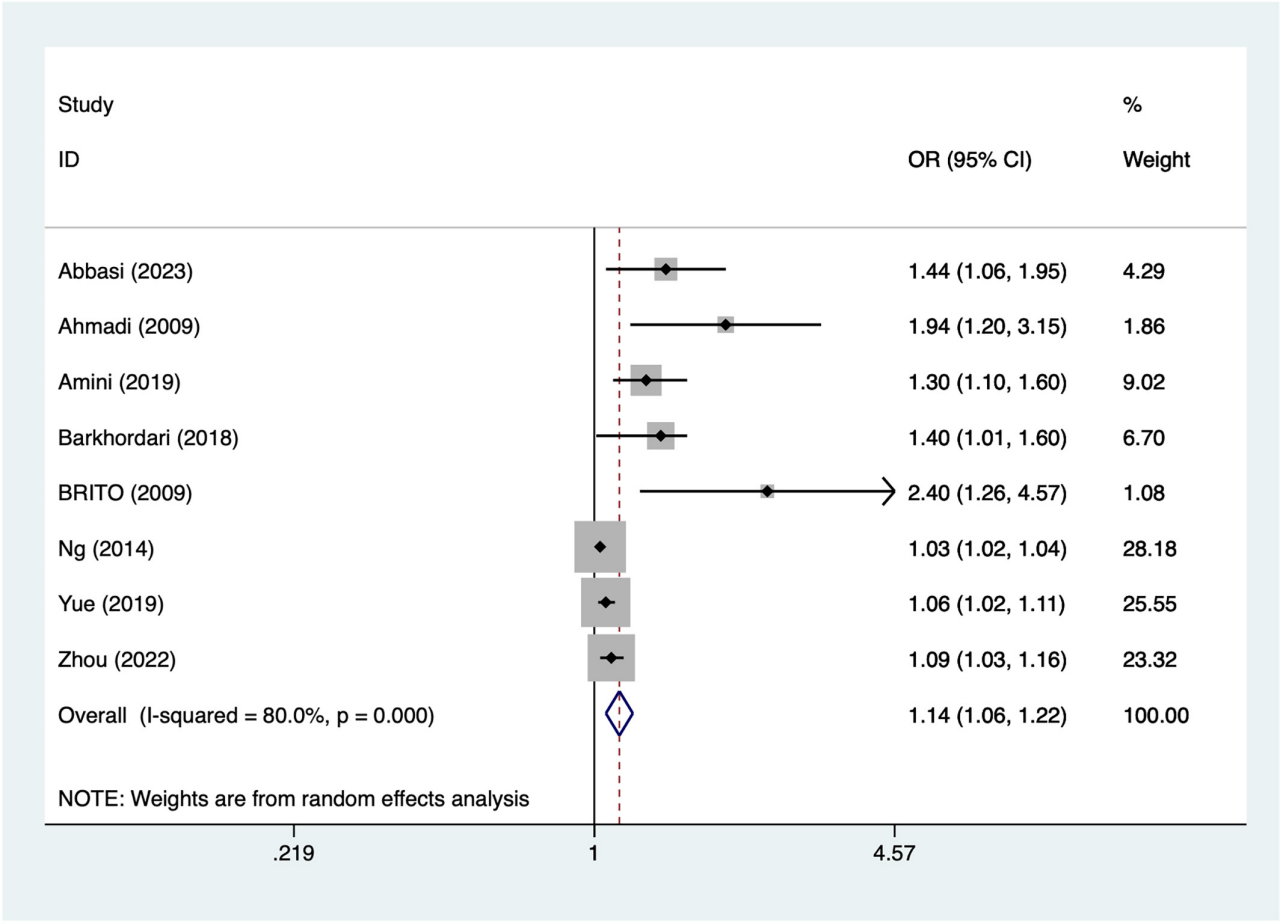
Forest plot of meta-analysis for prolonged cardiopulmonary bypass.

Eight articles mentioned prolonged cardiopulmonary bypass. Heterogeneity testing (I^2^ = 80.0%, *P* = 0.001) was conducted using a random-effects model. The results (Figure 3) suggest that longer bypass duration was associated with increased odds of postoperative AKI per hour of bypass [OR = 1.14, 95% CI (1.06, 1.22)]. Due to significant heterogeneity, sensitivity analysis was conducted by sequentially excluding individual studies. The results (Supplementary Figure 2) indicate that the indicator remains stable.

#### Diabetes

**FIGURE 4:**
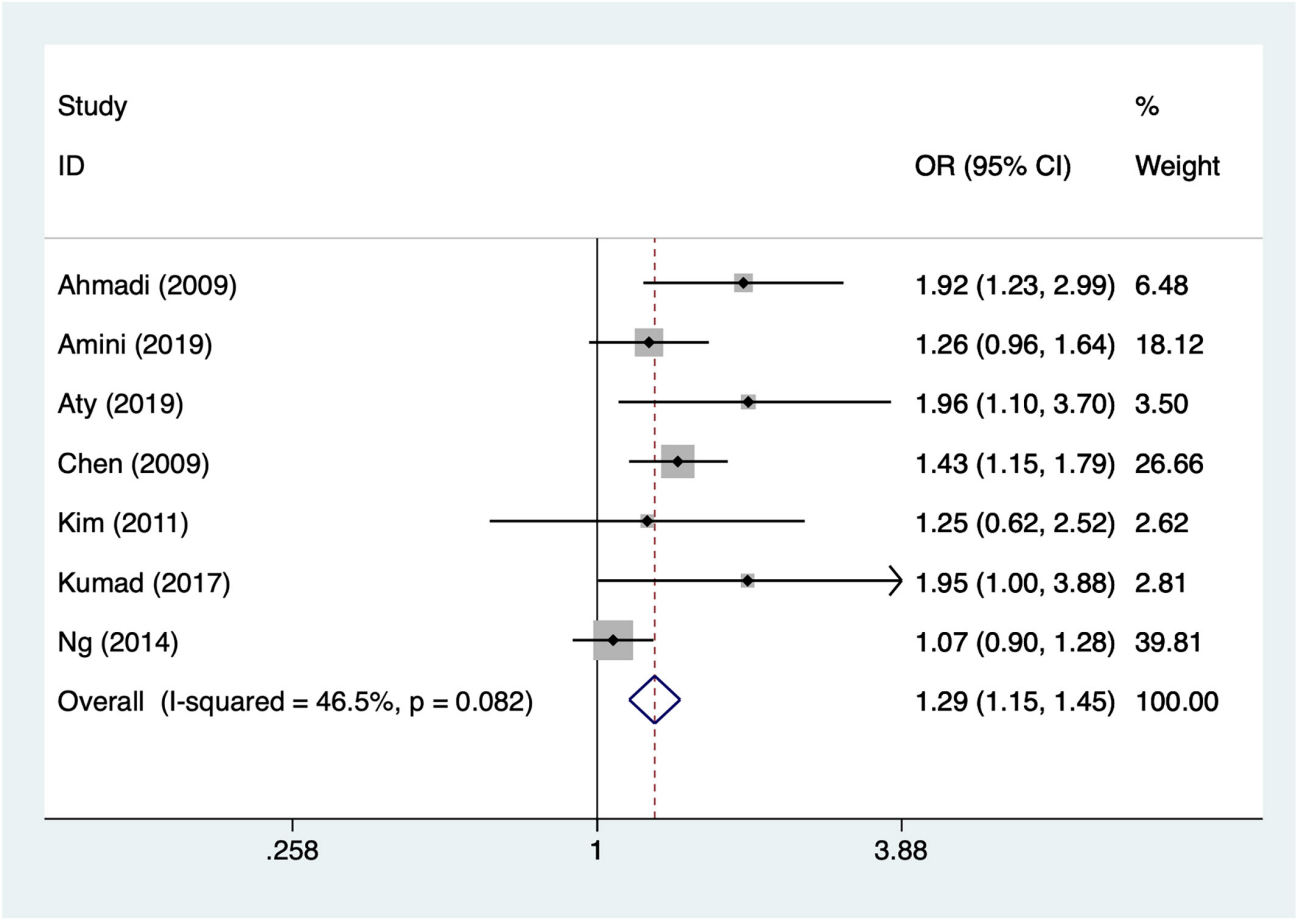
Forest plot of meta-analysis for diabetes.

Seven articles mentioned diabetes. Heterogeneity testing (I^2^ = 46.5%, *P* = 0.001) was conducted using a fixed-effects model. The results (Figure 4) suggest that diabetes may be associated with the occurrence of AKI after CABG [OR = 1.29, 95% CI (1.15, 1.45)].

#### Intra-aortic balloon pump

**FIGURE 5:**
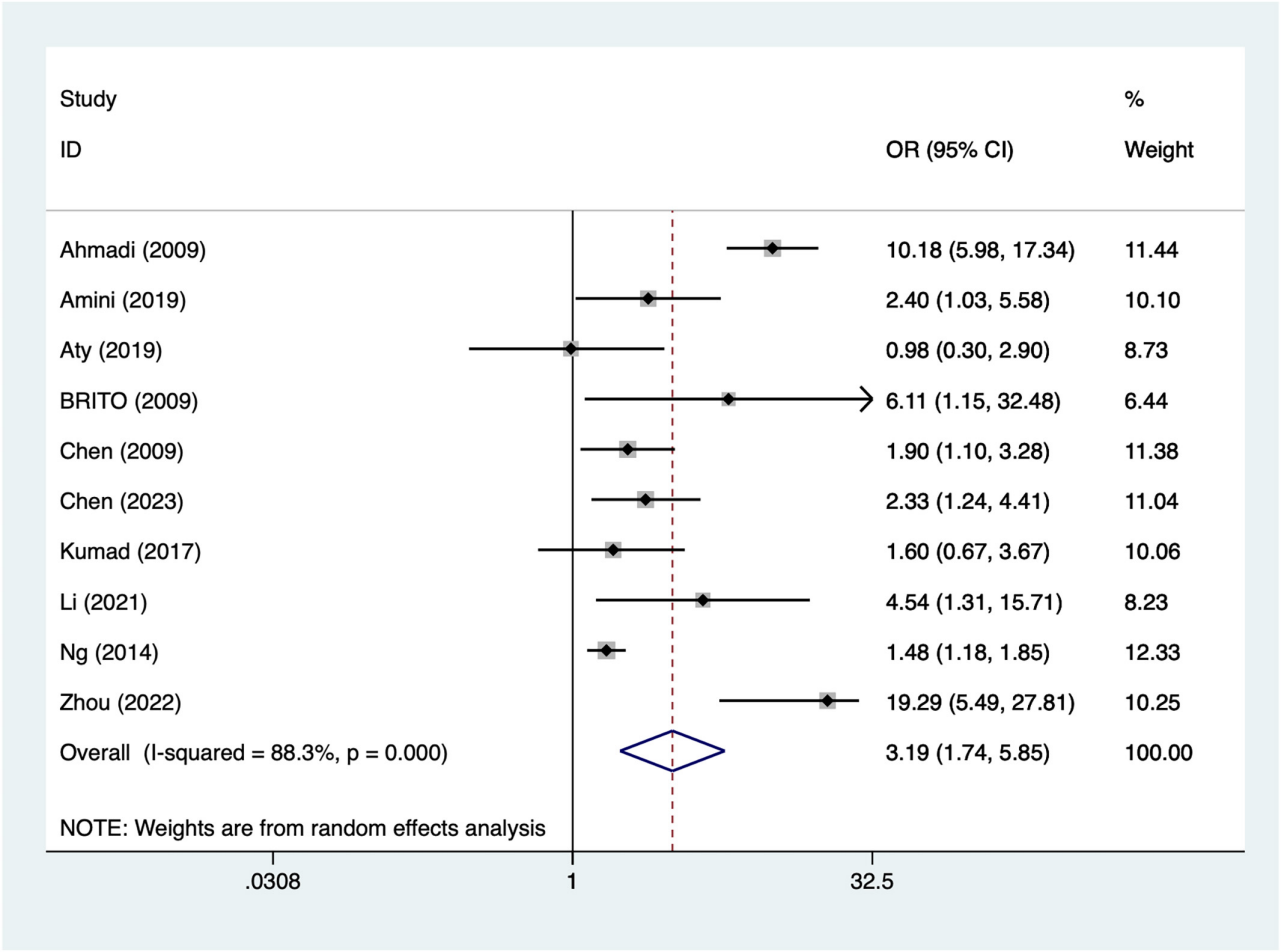
Forest plot of meta-analysis for intra-aortic balloon pump.

A total of 10 articles mentioned intra-aortic balloon pump. Heterogeneity testing (I^2^ = 88.3%, *P* = 0.001) was conducted using a random-effects model. The results (Figure 5) suggest that intra-aortic balloon pump may be associated with the occurrence of AKI after CABG [OR = 3.19, 95% CI (1.74, 5.85)]. Due to significant heterogeneity, sensitivity analysis was conducted by sequentially excluding individual studies. The results (Supplementary Figure 3) indicate that the indicator remains stable. When excluding Zhou’s (38) study results [OR = 2.58, 95% CI (1.50, 4.43)].

#### Transfusion of red blood cells

**FIGURE 6:**
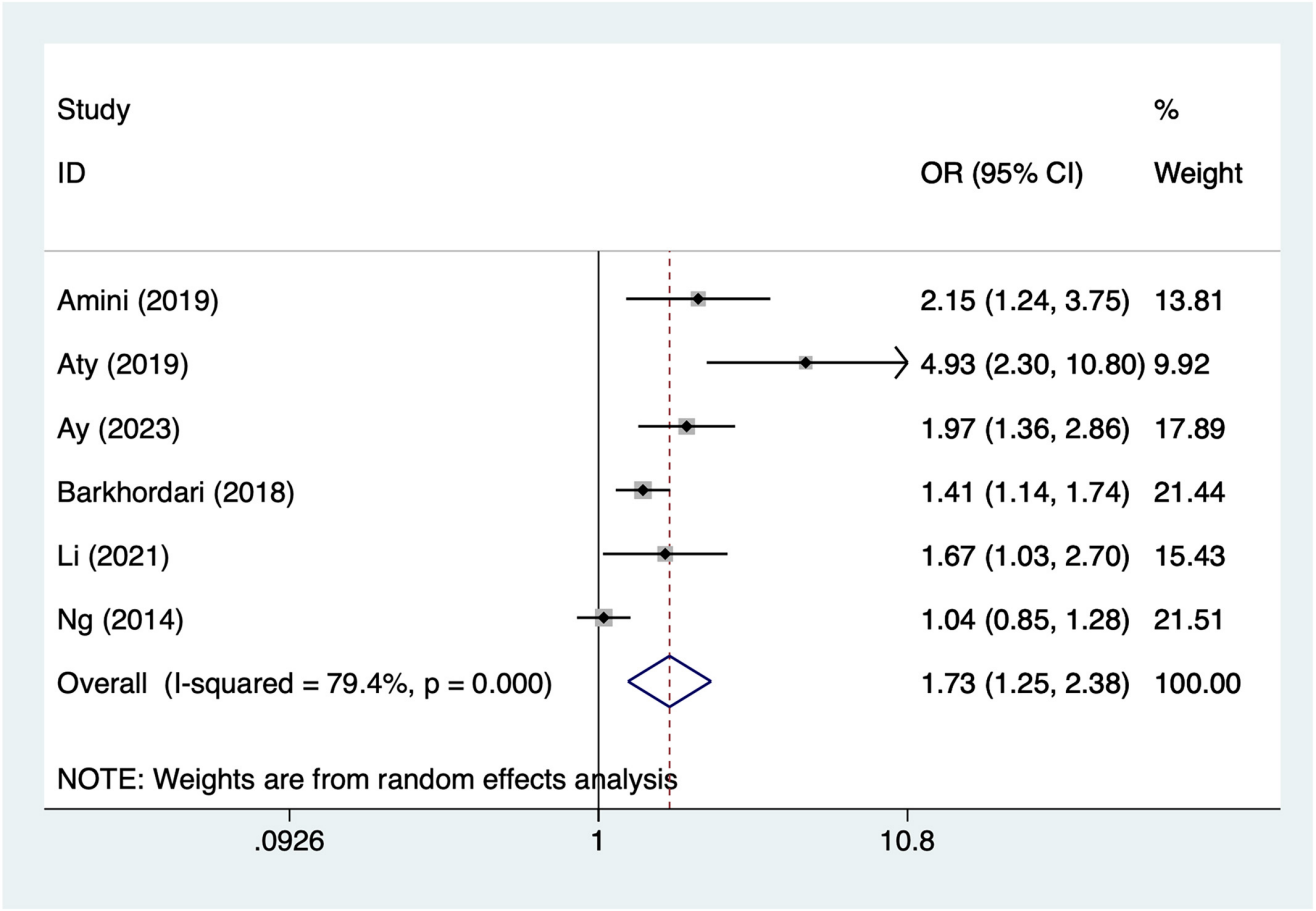
Forest plot of meta-analysis for transfusion of red blood cells.

A total of 10 articles mentioned transfusion of red blood cells. Heterogeneity testing (I^2^ = 79.4%, *P* = 0.001) was conducted using a random-effects model. The results (Figure 6) suggest that transfusion of red blood cells may be associated with the occurrence of AKI after CABG [OR = 1.73, 95% CI (1.25, 2.38)]. Due to significant heterogeneity, sensitivity analysis was conducted by sequentially excluding individual studies. The results (Supplementary Figure 4) indicate that the indicator remains stable.

### Publication bias

This study employed funnel plots and Egger’s test to detect publication bias. Results from funnel plots and Egger’s test (Supplementary Figures 5–9) indicated funnel plot asymmetry for older age (*P* = 0.001), prolonged cardiopulmonary bypass (*P* = 0.001), and transfusion of red blood cells (*P* = 0.025), suggesting potential publication bias. In contrast, funnel plots for diabetes (*P* = 0.17) and intra-aortic balloon pump (*P* = 0.77) showed symmetry, indicating a low likelihood of publication bias.

### Trim-and-fill results

Due to publication bias associated with older age, prolonged cardiopulmonary bypass, and red blood cell transfusion, the trim-and-fill method was employed to further assess the reliability of the results. The findings (Supplementary Figures 10–12) indicate that the conclusions remain robust even in the presence of publication bias.

### Meta- regression results

This study conducted a meta-regression analysis on Age, Prolonged cardiopulmonary bypass, Intra-aortic balloon pump, and Transfusion of red blood cells. The results (Supplementary Table 2) indicate that Year, Country, study design, and AKI diagnosis are not sources of heterogeneity for the above indicators.

## Discussion

In this study, we systematically evaluated variables associated with postoperative acute kidney injury following coronary artery bypass graft surgery through a meta-analysis of observational studies. Higher age, longer cardiopulmonary bypass duration, diabetes mellitus, intra-aortic balloon pump use, and red blood cell transfusion were all significantly associated with an increased likelihood of AKI. As all included studies were observational, these findings should be interpreted as associations rather than causal relationships. Nevertheless, they provide clinically relevant information for identifying patients at elevated risk of AKI.

This study indicates that advanced age may increase the risk of AKI following CABG. This finding aligns with previous research (39), suggesting that elderly patients may be more susceptible to AKI due to physiological decline, reduced renal reserve capacity, and increased perioperative and postoperative complications. Elderly patients often present with multiple comorbidities, such as hypertension and diabetes, which may further exacerbate renal burden (40). Additionally, older patients may experience issues with drug metabolism and renal clearance during postoperative recovery (14). Therefore, developing individualized postoperative care plans for elderly patients to reduce AKI incidence represents a critical direction for future research and clinical practice. Prolonged cardiopulmonary bypass duration is significantly associated with the occurrence of postoperative AKI following CABG. Extended bypass time may lead to inadequate renal perfusion and reduced oxygen supply, thereby contributing to the development of acute kidney injury (41). During cardiopulmonary bypass, hemodynamic fluctuations, variations in oxygenation levels, and the use of bypass machines may cause direct or indirect renal damage. Additionally, prolonged bypass duration may exacerbate intraoperative inflammatory responses, representing another potential mechanism for AKI development (42). To mitigate the adverse effects of cardiopulmonary bypass on renal function, future strategies should focus on shortening bypass duration and optimizing intraoperative hemodynamic management. Diabetes is one of the major risk factors for postoperative AKI following CABG. Patients with diabetes often exhibit microvascular complications and impaired renal perfusion, which may increase the risk of postoperative AKI (43). The hyperglycemic state in diabetic patients can also trigger oxidative stress and inflammatory responses, further exacerbating renal damage (44). Clinically, blood glucose control in diabetic patients should be more stringent to avoid fluctuations in blood glucose levels before and during surgery, thereby reducing the incidence of AKI. The use of intra-aortic balloon pump may be associated with the occurrence of AKI. Intra-aortic balloon pump is commonly used to support patients with cardiac dysfunction, but its use may lead to adverse effects such as fluid accumulation, blood pressure fluctuations, and impaired renal perfusion (45). The impact of intra-aortic balloon pump on postoperative AKI may be closely related to hemodynamic changes and impaired renal perfusion occurring during its operation. Therefore, renal function should be closely monitored during intra-aortic balloon pump therapy, and appropriate measures should be promptly implemented to prevent AKI (46). One study by Zhou et al. (38) reported an exceptionally high odds ratio (OR = 19.29, 95% CI: 5.49–27.81) for intra-aortic balloon pump use and postoperative AKI, contributing 10.25% weight in the pooled analysis. This study specifically included a high-risk population with severe cardiac dysfunction, which likely explains the elevated effect estimate. To ensure that the overall findings were not driven by this single study, we conducted a sensitivity analysis excluding Zhou et al. (38). The pooled OR for IABP remained significant [OR = 2.58, 95% CI (1.50, 4.43)], indicating that the main results are robust and not overly influenced by this outlier. Red blood cell transfusion is significantly associated with the occurrence of postoperative AKI following CABG. The reasons for red blood cell transfusion are typically related to excessive intraoperative blood loss, hypovolemia, and anemia (47). Transfusion may trigger the release of cytokines and inflammatory mediators, leading to acute kidney injury. Red blood cell transfusion may also increase the risk of infection, further complicating the postoperative recovery process (48). Therefore, during CABG surgery, unnecessary transfusions should be minimized, and more precise blood management strategies should be adopted to prevent the occurrence of AKI.

In this study, we employed funnel plots and Egger’s test to assess the presence of publication bias. Results indicated publication bias existed in studies examining age, cardiopulmonary bypass duration, and red blood cell transfusion, whereas studies on diabetes and intra-aortic balloon pump showed no evidence of publication bias. The presence of publication bias may stem from unpublished negative results or the exclusion of small-sample studies from analysis. To enhance the reliability of findings, future studies should further strengthen the investigation of these factors and adopt more rigorous research designs and methodologies to mitigate the impact of publication bias.

In this study, substantial heterogeneity was observed across several analyses (e.g., age I^2^ = 83.6%, cardiopulmonary bypass duration I^2^ = 80.0%, IABP I^2^ = 88.3%). The sources of this heterogeneity likely include differences in study populations, variations in definitions of AKI, differences in surgical techniques, and perioperative management practices. For example, some studies included exclusively high-risk or elderly patients, while others had broader inclusion criteria. Variability in cardiopulmonary bypass protocols, transfusion thresholds, and postoperative care may also contribute to the observed heterogeneity. Despite these differences, sensitivity analyses confirmed the robustness of the pooled estimates, suggesting that the identified associations are reliable.

## Clinical implications

The findings of this study have several practical implications for perioperative management of patients undergoing CABG. First, preoperative optimization of high-risk patients, including careful assessment of renal function, glycemic control in diabetic patients, and management of comorbidities, may help reduce postoperative AKI risk. Second, intraoperative perfusion strategies, such as minimizing cardiopulmonary bypass duration, maintaining stable hemodynamics, and ensuring adequate renal perfusion, could mitigate renal injury. Finally, blood management strategies, including judicious use of red blood cell transfusions and optimization of hemoglobin levels, may further decrease the likelihood of AKI. Incorporating these strategies into clinical practice may improve postoperative outcomes and provide individualized care for patients at elevated risk.

## Strengths and limitations

The strength of this study lies in its systematic and comprehensive approach. By integrating multiple studies on risk factors for AKI following CABG, it conducted an in-depth analysis of the relationship between various clinical variables—such as age, cardiopulmonary bypass duration, and diabetes—and AKI. This ensures the findings are not only comprehensive but also possess high clinical reference value. Furthermore, the use of a random-effects model for meta-analysis, coupled with sensitivity analyses, ensures robust and reliable results. Consistent trends across all examined variables provide strong support for clinical assessment and management of postoperative AKI following CABG.

However, certain limitations remain. First, most of the included studies were retrospective, posing a risk of bias. Second, discrepancies in AKI definitions, patient selection criteria, and study designs across different investigations may have contributed to inconsistent findings. Although sensitivity analyses were conducted to assess the robustness of individual indicators, potential confounding factors could not be entirely ruled out. Finally, the small sample sizes in some studies may have compromised the accuracy and generalizability of the results.

## Future research directions

Future studies should further validate the impact of these risk factors on AKI occurrence through large-scale, prospective, randomized controlled trials and explore potential intervention strategies. Additionally, research should focus on individualized risk assessment and precision treatment for patients, adopting a multidisciplinary collaborative approach that integrates hemodynamic monitoring and intraoperative drug regulation to minimize AKI incidence as much as possible.

## Conclusion

In conclusion, this systematic review and meta-analysis identified several clinical variables—advanced age, prolonged cardiopulmonary bypass duration, diabetes mellitus, intra-aortic balloon pump use, and red blood cell transfusion—that are significantly associated with postoperative AKI following CABG. Although publication bias and study heterogeneity exist, these findings provide valuable clinical guidance for risk assessment and perioperative management. Future research should prioritize well-designed, high-quality prospective studies using standardized definitions of AKI to validate these associations and develop effective preventive and management strategies for patients undergoing CABG.

## Data Availability

The datasets presented in this study can be found in online repositories. The names of the repository/repositories and accession number(s) can be found in the article/Supplementary material.
