# Supplementary material for: Risk factors for acute kidney injury after coronary artery bypass graft surgery: a systematic review and meta-analysis
Source: Front Med (Lausanne). 2026 Feb 24;13:1722801. doi: 10.3389/fmed.2026.1722801 (PMC12971401; doi:10.3389/fmed.2026.1722801)
Supplement: Supplementary file 1 [file Data_Sheet_1.docx]

Table S1 specific search strategy

((("Coronary Artery Bypass"[Mesh]) OR (((((((((((((Coronary Artery Bypass[Title/Abstract]) OR (Artery Bypass, Coronary[Title/Abstract])) OR (Artery Bypasses, Coronary[Title/Abstract])) OR (Bypasses, Coronary Artery[Title/Abstract])) OR (Coronary Artery Bypasses[Title/Abstract])) OR (Coronary Artery Bypass Grafting[Title/Abstract])) OR (Coronary Artery Bypass Surgery[Title/Abstract])) OR (Aortocoronary Bypass[Title/Abstract])) OR (Aortocoronary Bypasses[Title/Abstract])) OR (Bypass, Aortocoronary[Title/Abstract])) OR (Bypasses, Aortocoronary[Title/Abstract])) OR (Bypass Surgery, Coronary Artery[Title/Abstract])) OR (Bypass, Coronary Artery[Title/Abstract]))) AND (("Acute Kidney Injury"[Mesh]) OR ((((((((((((((((((((((((Acute Kidney Injury[Title/Abstract]) OR (Acute Kidney Injuries[Title/Abstract])) OR (Kidney Injuries, Acute[Title/Abstract])) OR (Kidney Injury, Acute[Title/Abstract])) OR (Acute Renal Injury[Title/Abstract])) OR (Acute Renal Injuries[Title/Abstract])) OR (Renal Injuries, Acute[Title/Abstract])) OR (Renal Injury, Acute[Title/Abstract])) OR (Kidney Failure, Acute[Title/Abstract])) OR (Acute Kidney Failures[Title/Abstract])) OR (Kidney Failures, Acute[Title/Abstract])) OR (Acute Kidney Failure[Title/Abstract])) OR (Acute Renal Failure[Title/Abstract])) OR (Acute Renal Failures[Title/Abstract])) OR (Renal Failures, Acute[Title/Abstract])) OR (Renal Failure, Acute[Title/Abstract])) OR (Renal Insufficiency, Acute[Title/Abstract])) OR (Acute Renal Insufficiencies[Title/Abstract])) OR (Renal Insufficiencies, Acute[Title/Abstract])) OR (Acute Kidney Insufficiency[Title/Abstract])) OR (Acute Renal Insufficiency[Title/Abstract])) OR (Kidney Insufficiency, Acute[Title/Abstract])) OR (Acute Kidney Insufficiencies[Title/Abstract])) OR (Kidney Insufficiencies, Acute[Title/Abstract])))) AND (("Risk Factors"[Mesh]) OR (((((((((((((((((((Risk Factors[Title/Abstract]) OR (Factor, Risk[Title/Abstract])) OR (Risk Factor[Title/Abstract])) OR (Population at Risk[Title/Abstract])) OR (Populations at Risk[Title/Abstract])) OR (Risk Scores[Title/Abstract])) OR (Risk Score[Title/Abstract])) OR (Score, Risk[Title/Abstract])) OR (Risk Factor Scores[Title/Abstract])) OR (Risk Factor Score[Title/Abstract])) OR (Score, Risk Factor[Title/Abstract])) OR (Health Correlates[Title/Abstract])) OR (Correlates, Health[Title/Abstract])) OR (Social Risk Factors[Title/Abstract])) OR (Factor, Social Risk[Title/Abstract])) OR (Factors, Social Risk[Title/Abstract])) OR (Risk Factor, Social[Title/Abstract])) OR (Risk Factors, Social[Title/Abstract])) OR (Social Risk Factor[Title/Abstract])))

Table S2 meta regression results

| Risk factors | Year | Country | study design | AKI diagnosis |
| --- | --- | --- | --- | --- |
| Age | 0.54 | 0.09 | 0.13 | 0.25 |
| Prolonged cardiopulmonary bypass | 0.16 | 0.19 | 0.28 | 0.49 |
| In-tra-aortic balloon pump | 0.75 | 0.69 | 0.41 | 0.18 |
| Transfusion of red blood cells | 0.28 | 0.37 | 0.62 | 0.77 |


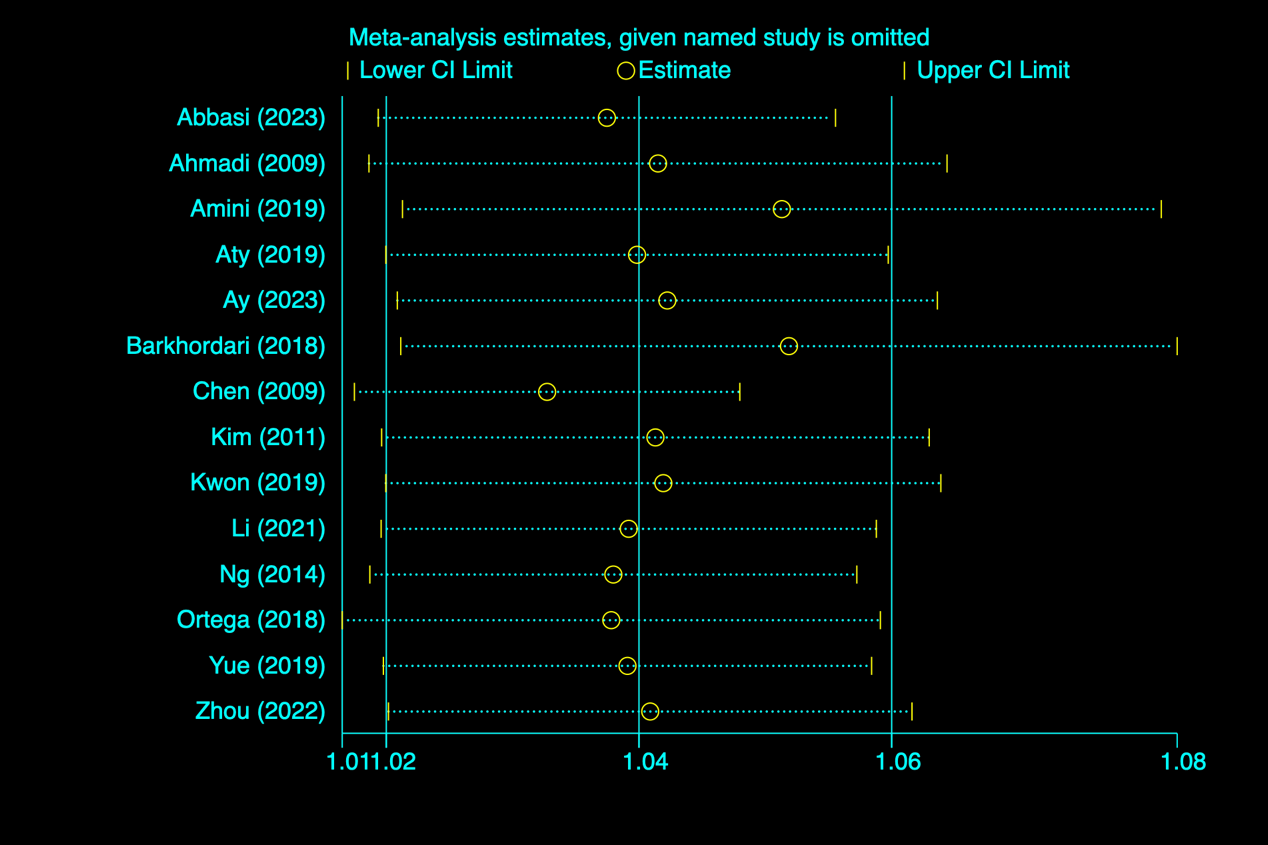


Figure s1 Sensitivity analysis plot of age


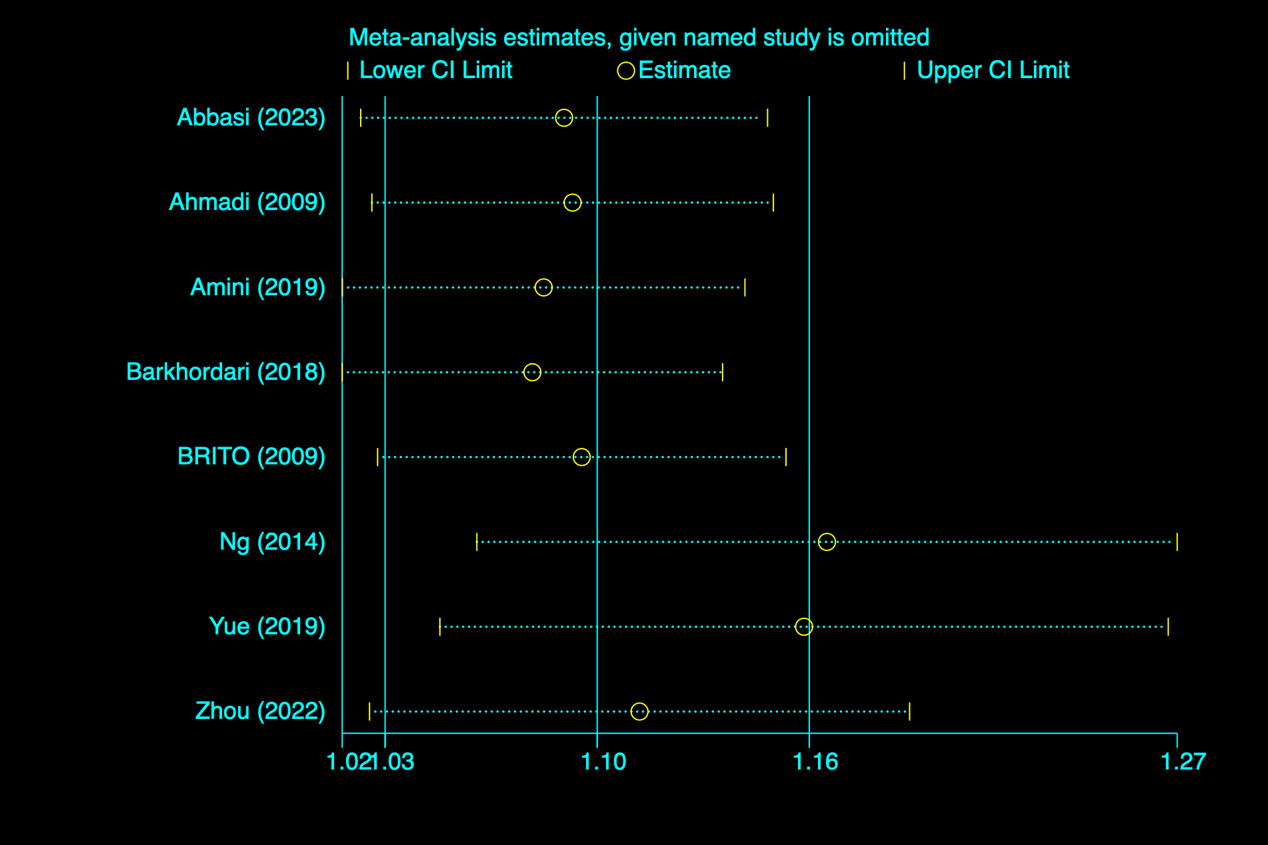


Figure s2 Sensitivity analysis plot of prolonged cardiopulmonary bypass


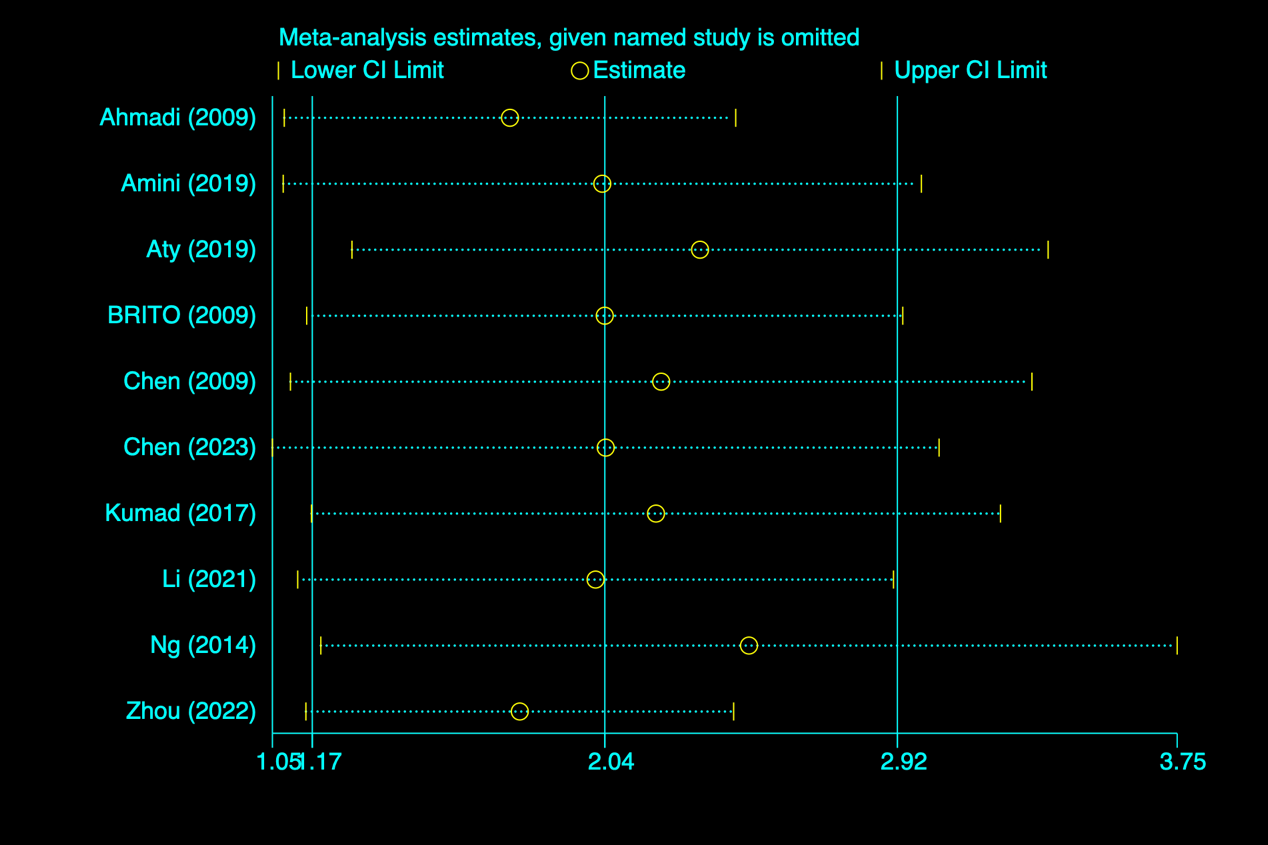


Figure s3 Sensitivity analysis plot of in- tra-aortic balloon pump


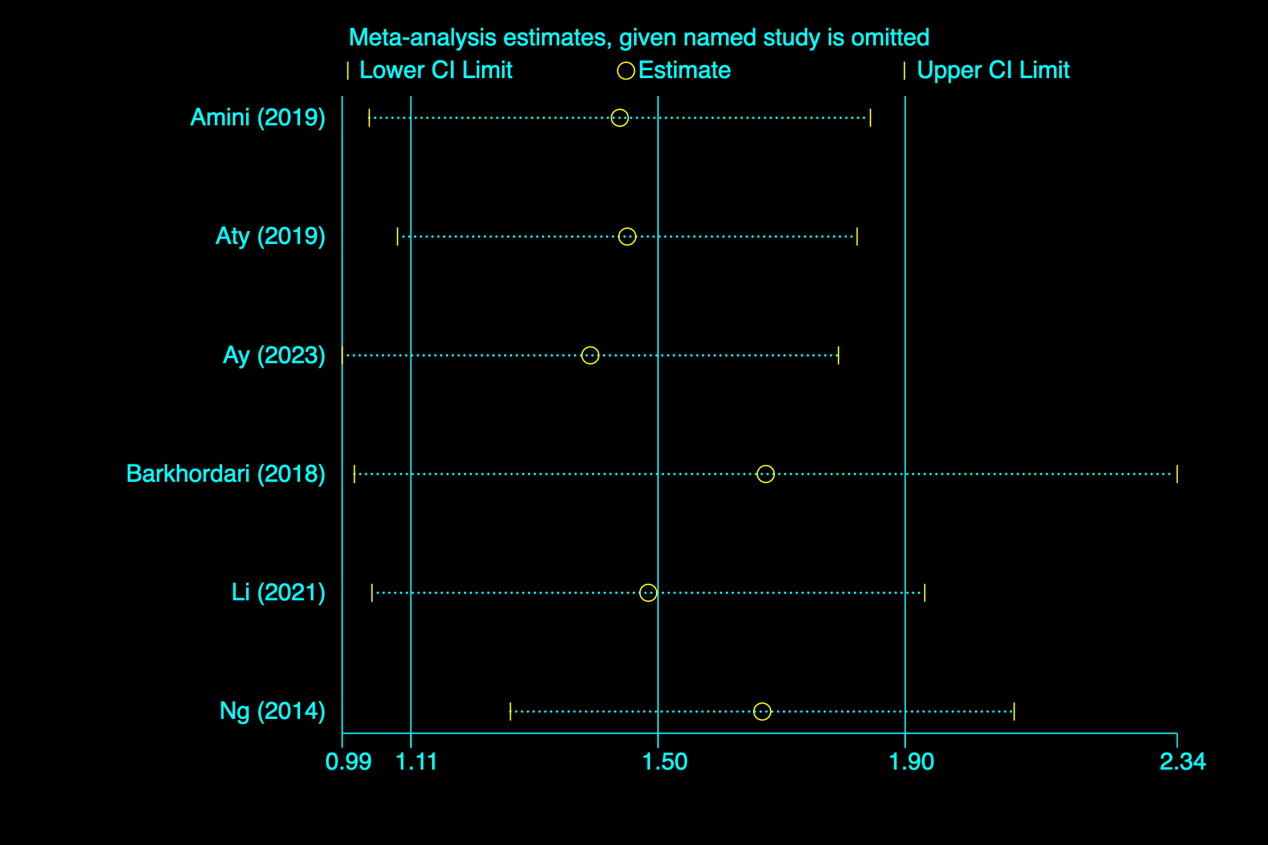


Figure s4 Sensitivity analysis plot of transfusion of red blood cells


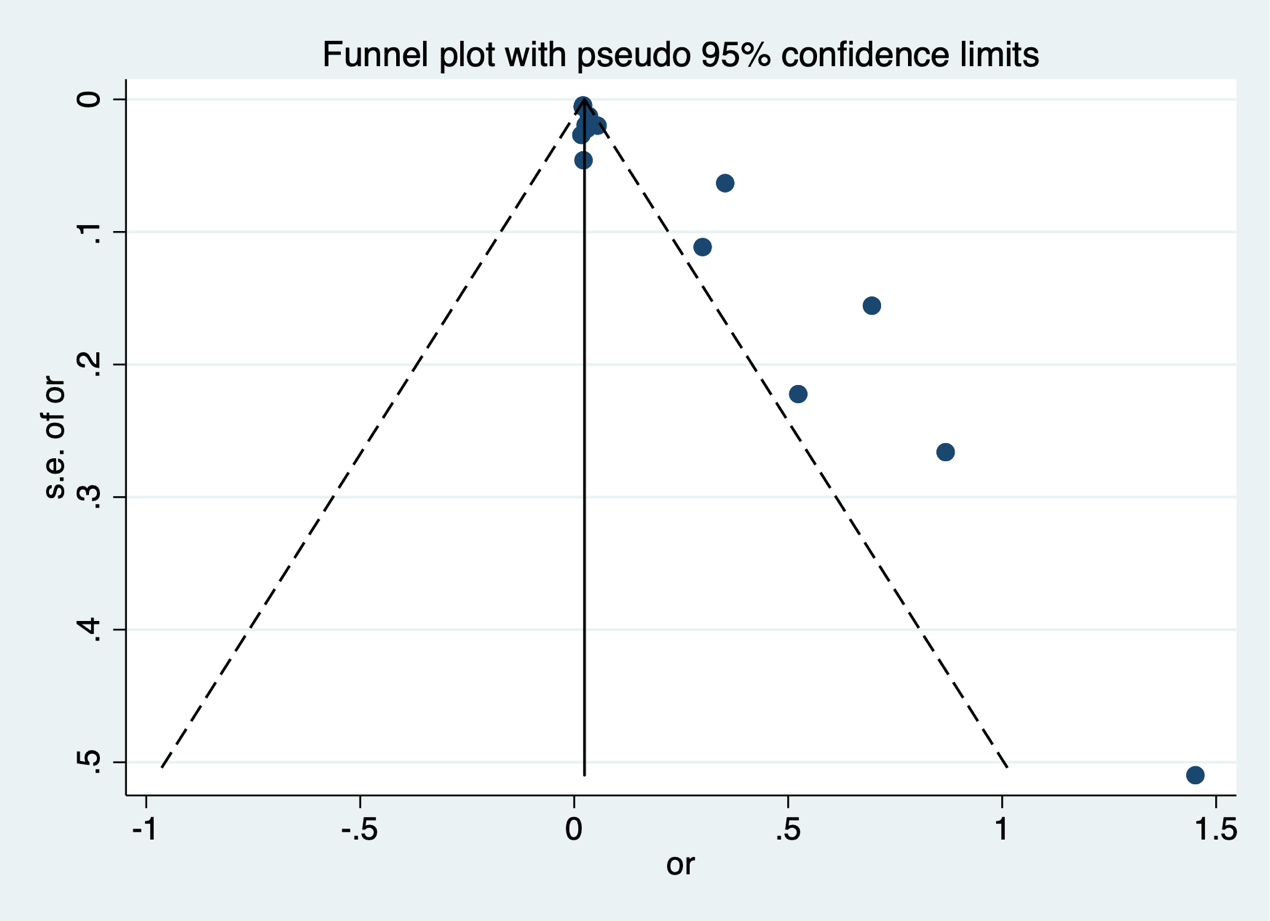


Figure S5 Funnel plot of meta-analysis of age


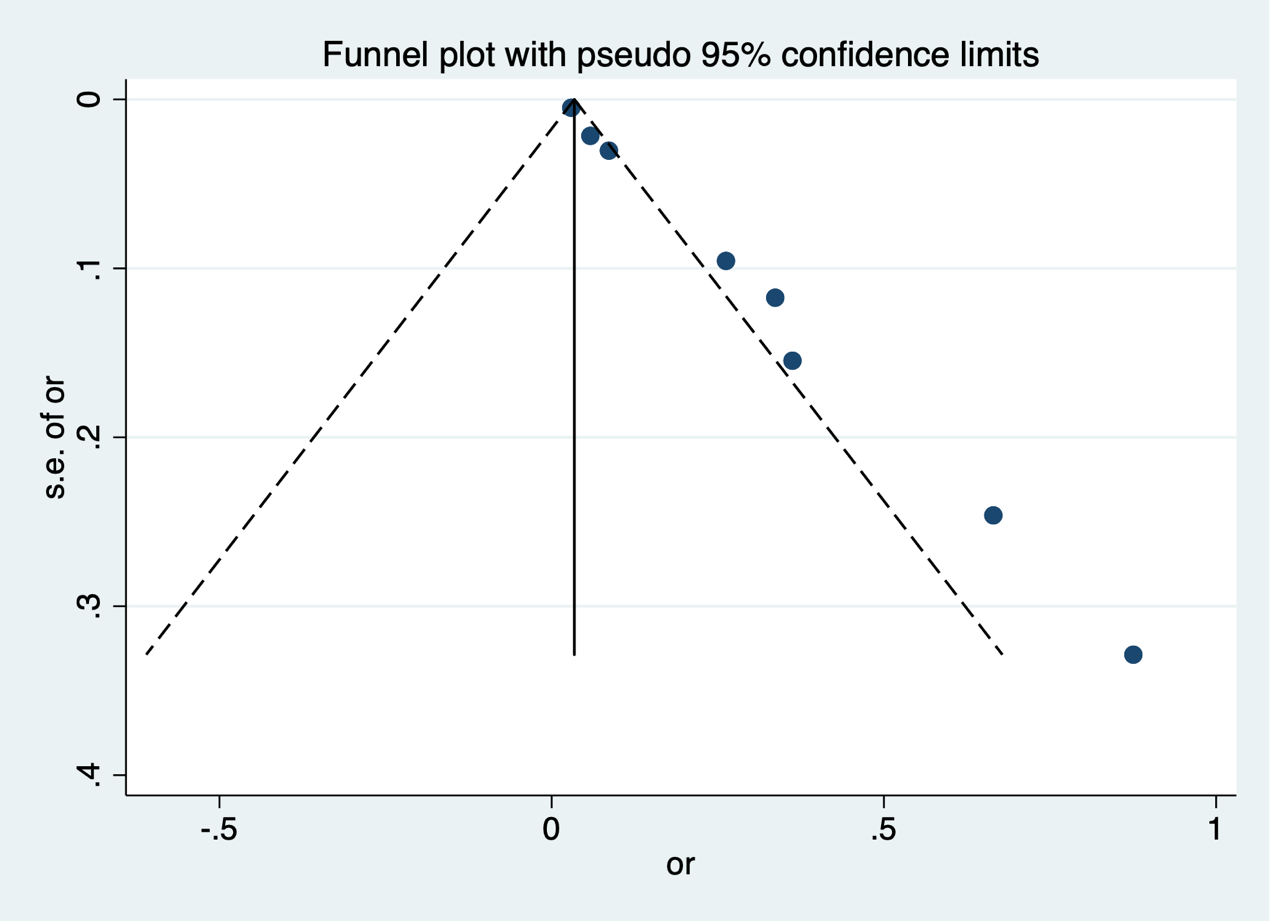


Figure S6 Funnel plot of meta-analysis of prolonged cardiopulmonary bypass


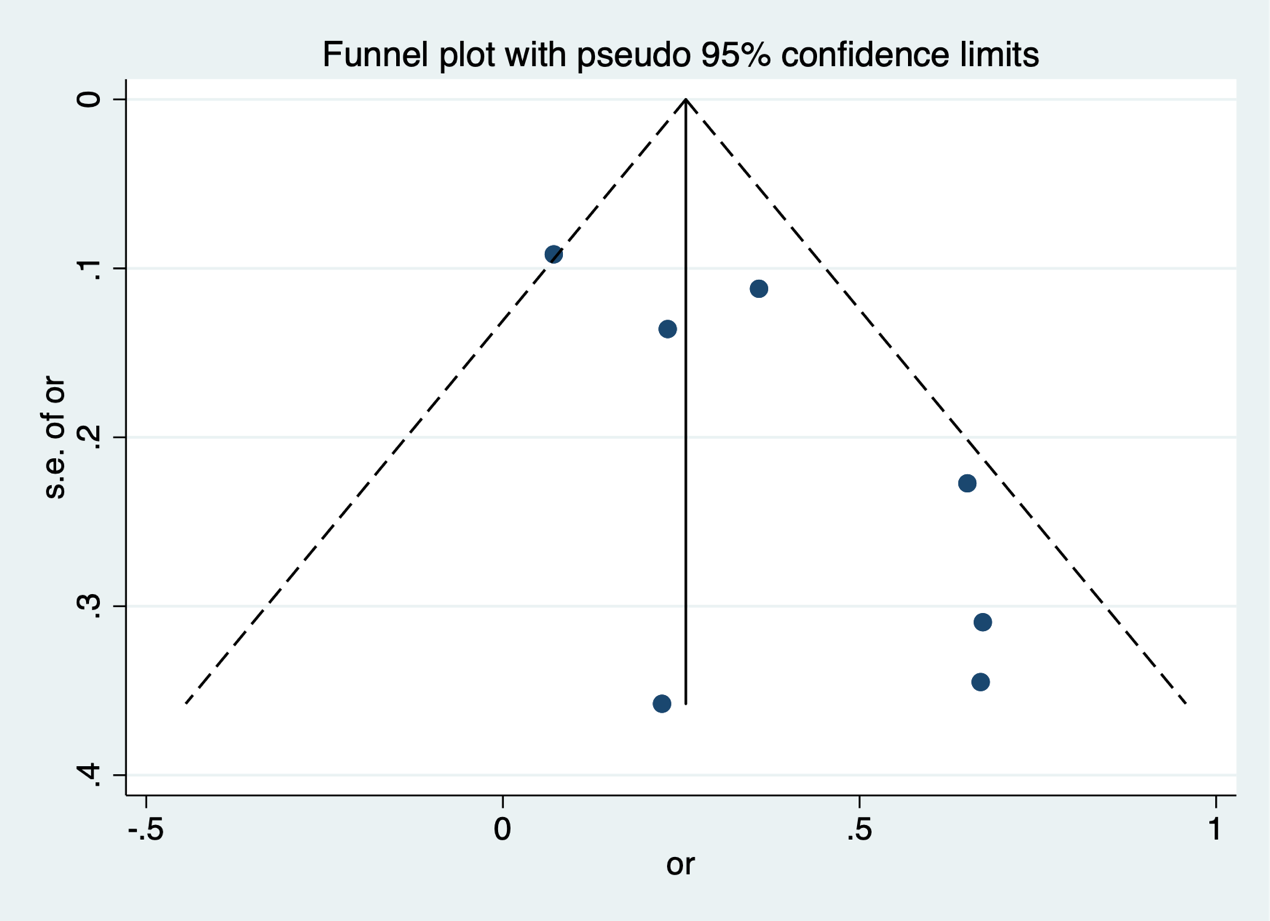


Figure S7 Funnel plot of meta-analysis of diabetes


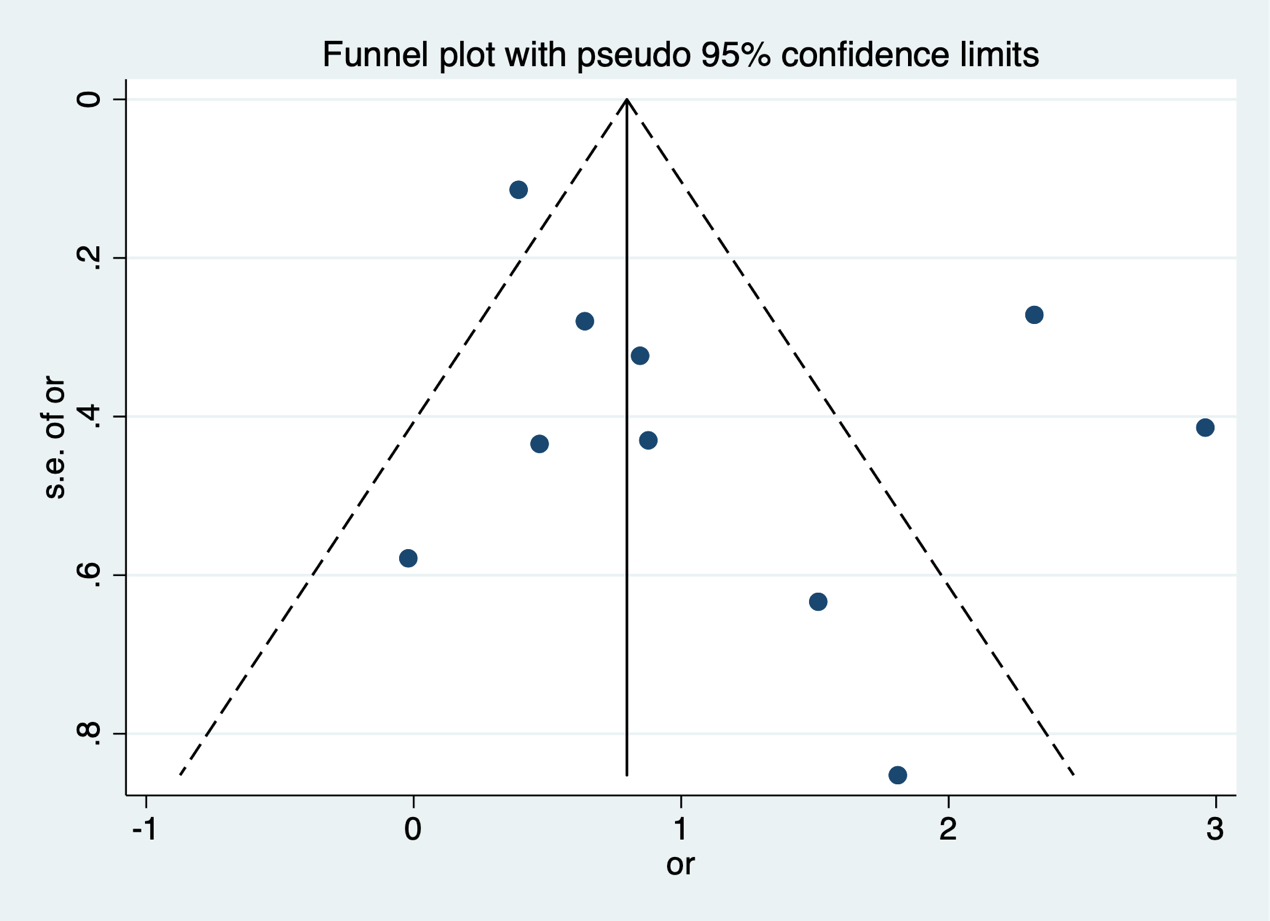


Figure S8 Funnel plot of meta-analysis of in- tra-aortic balloon pump


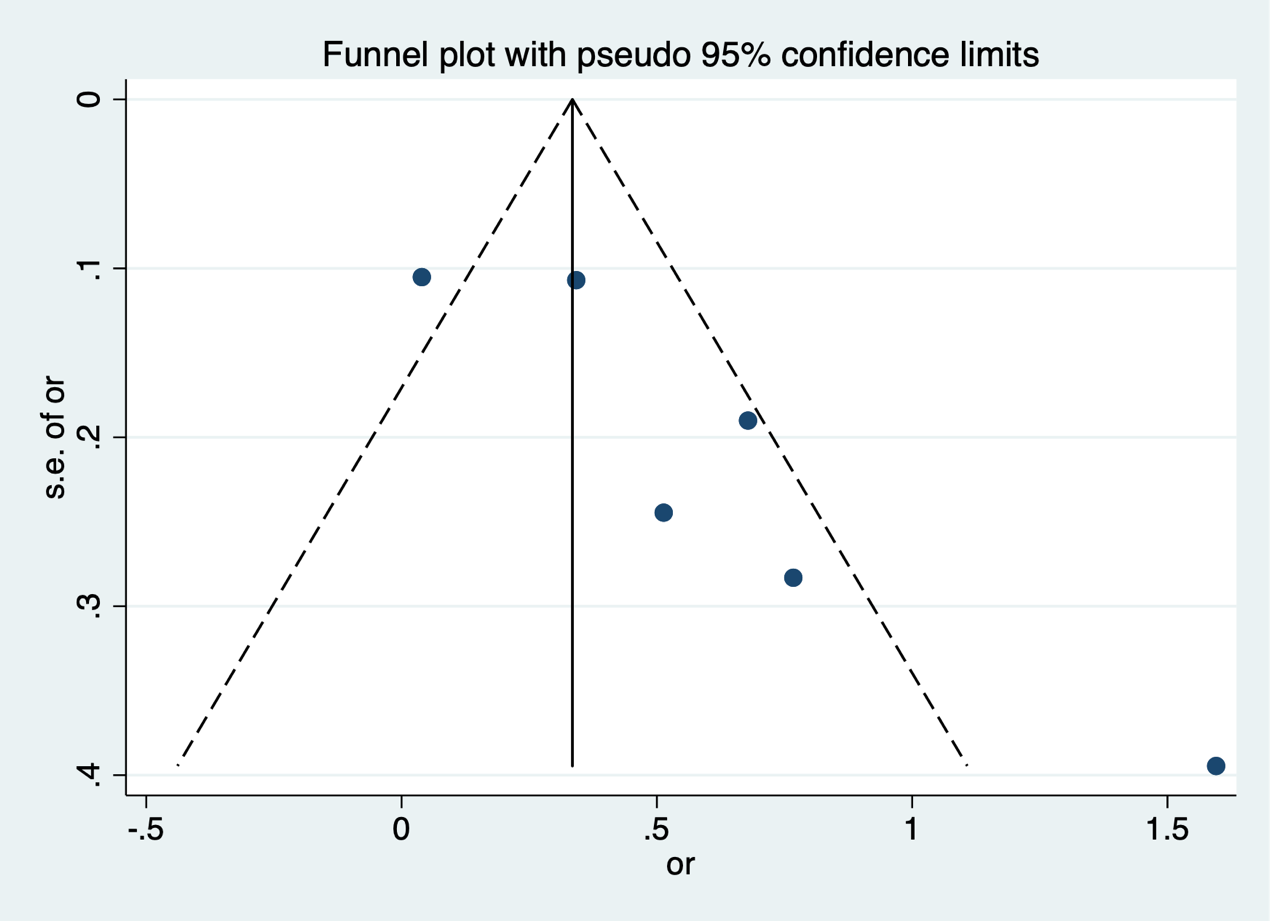


Figure S9 Funnel plot of meta-analysis of transfusion of red blood cells


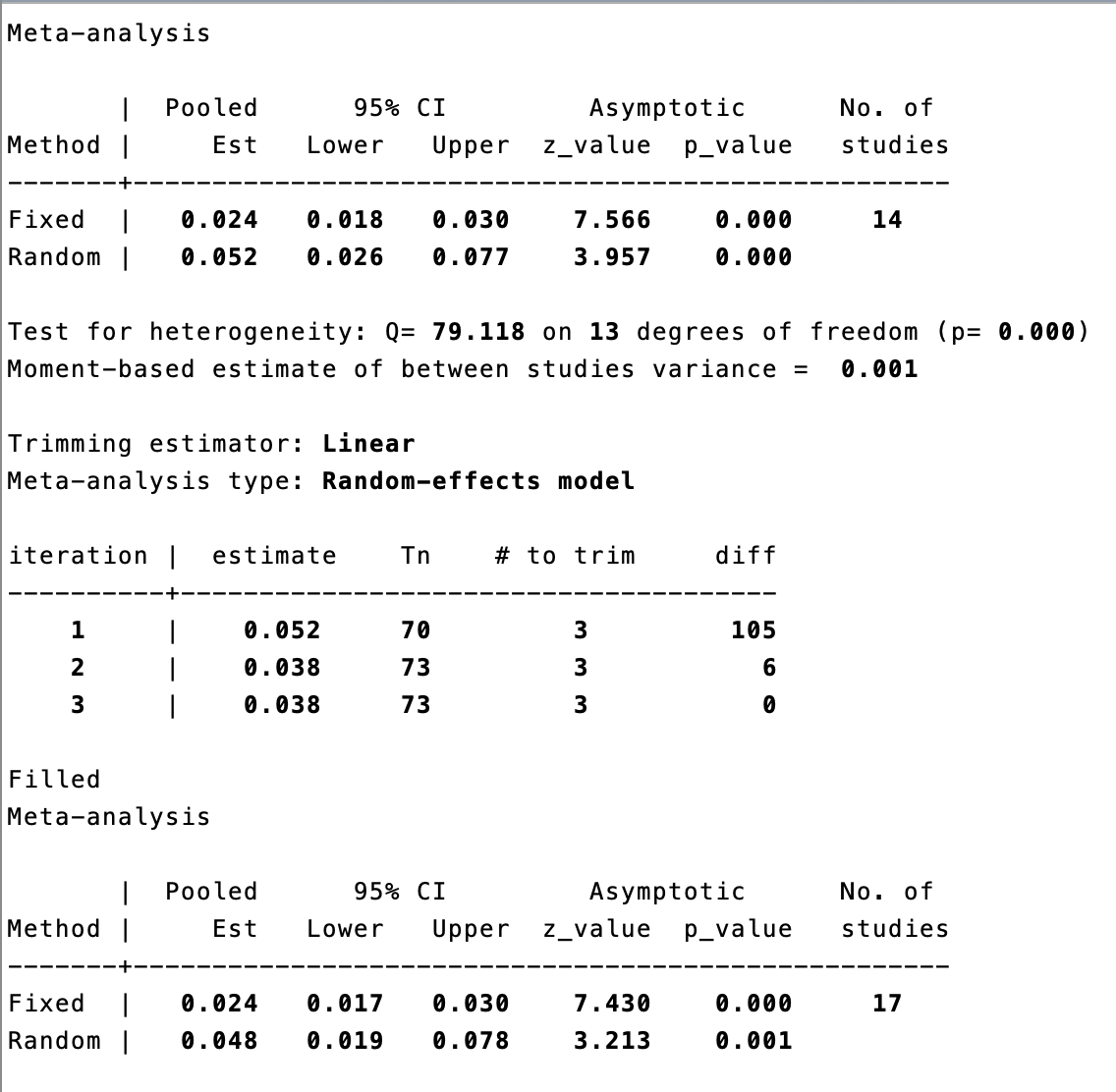


Figure S10 Results of Meta trim-and-fill for age.


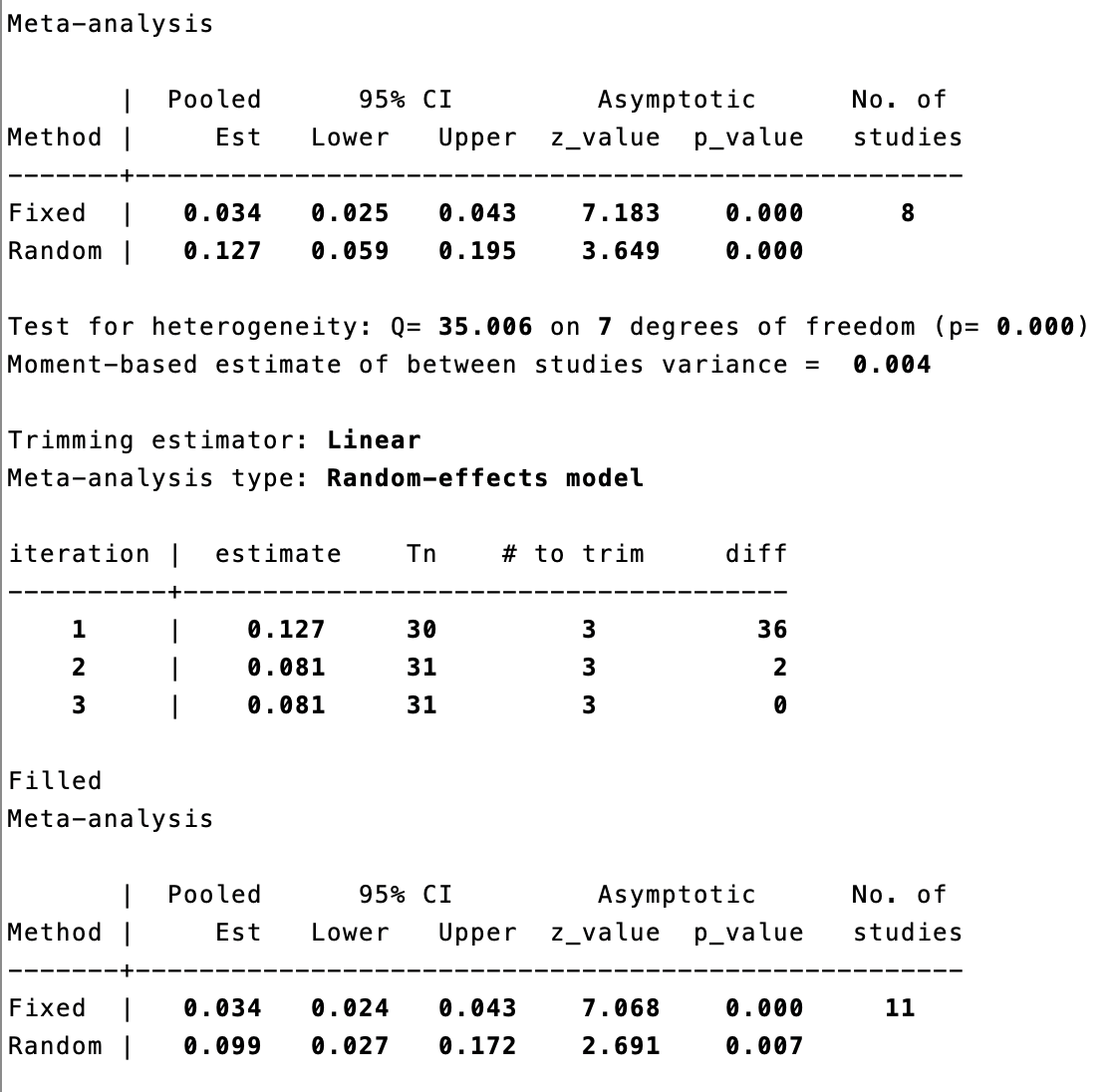


Figure S11 Results of Meta trim-and-fill for prolonged cardiopulmonary bypass


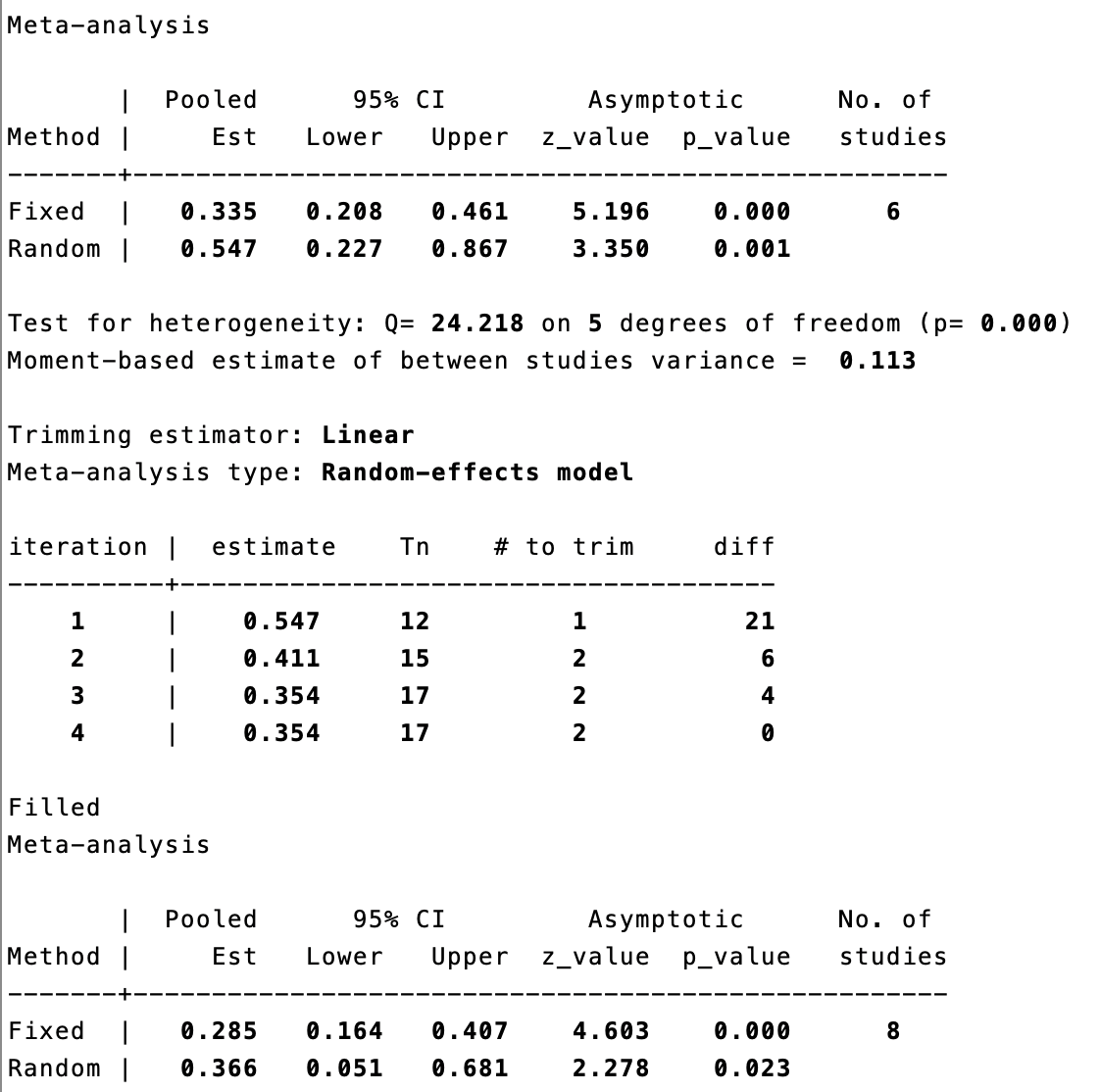


Figure S12 Results of Meta trim-and-fill for red blood cell transfusion
